# Postpartum plasma metabolomic profile among women with preeclampsia and preterm delivery: implications for long-term health

**DOI:** 10.1186/s12916-020-01741-4

**Published:** 2020-10-13

**Authors:** Xiumei Hong, Boyang Zhang, Liming Liang, Yan Zhang, Yuelong Ji, Guoying Wang, Hongkai Ji, Clary B. Clish, Irina Burd, Colleen Pearson, Barry Zuckerman, Frank B. Hu, Xiaobin Wang

**Affiliations:** 1 Center on the Early Life Origins of Disease, Department of Population, Family and Reproductive Health, Johns Hopkins University Bloomberg School of Public Health, 615 N. Wolfe St, Baltimore, MD 21205-2179 USA; 2 Department of Biostatistics, Johns Hopkins University Bloomberg School of Public Health, 615 N. Wolfe St, Baltimore, MD 21205 USA; 3 Department of Epidemiology, Harvard T.H. Chan School of Public Health, Boston, MA USA; 4 Department of Biostatistics, Harvard T.H. Chan School of Public Health, Boston, MA USA; 5 Broad Institute of MIT and Harvard University, Cambridge, MA USA; 6 Integrated Research Center for Fetal Medicine, Department of Gynecology and Obstetrics, Johns Hopkins University School of Medicine, Baltimore, MD 21287 USA; 7 Department of Pediatrics, Boston University School of Medicine and Boston Medical Center, Boston, MA 02118 USA; 8 Department of Nutrition, Harvard T.H. Chan School of Public Health, Boston, MA USA; 9 Channing Division of Network Medicine, Department of Medicine, Brigham and Women’s Hospital and Harvard Medical School, Boston, MA USA; 10 Division of General Pediatrics & Adolescent Medicine, Department of Pediatrics, Johns Hopkins University School of Medicine, Baltimore, MD 21205 USA

**Keywords:** Preterm delivery, Preeclampsia, Medically indicated preterm delivery, Spontaneous preterm delivery, Postpartum, Metabolome

## Abstract

**Background:**

Preeclampsia and preterm delivery (PTD) are believed to affect women’s long-term health including cardiovascular disease (CVD), but the biological underpinnings are largely unknown. We aimed to test whether maternal postpartum metabolomic profiles, especially CVD-related metabolites, varied according to PTD subtypes with and without preeclampsia, in a US urban, low-income multi-ethnic population.

**Methods:**

This study, from the Boston Birth Cohort, included 980 women with term delivery, 79 with medically indicated PTD (mPTD) and preeclampsia, 52 with mPTD only, and 219 with spontaneous PTD (sPTD). Metabolomic profiling in postpartum plasma was conducted by liquid chromatography-mass spectrometry. Linear regression models were used to assess the associations of each metabolite with mPTD with preeclampsia, mPTD only, and sPTD, respectively, adjusting for pertinent covariates. Weighted gene coexpression network analysis was applied to investigate interconnected metabolites associated with the PTD/preeclampsia subgroups. Bonferroni correction was applied to account for multiple testing.

**Results:**

A total of 380 known metabolites were analyzed. Compared to term controls, women with mPTD and preeclampsia showed a significant increase in 36 metabolites, mainly representing acylcarnitines and multiple classes of lipids (diacylglycerols, triacylglycerols, phosphocholines, and lysophosphocholines), as well as a decrease in 11 metabolites including nucleotides, steroids, and cholesteryl esters (CEs) (*P* < 1.3 × 10^−4^). Alterations of diacylglycerols, triacylglycerols, and CEs in women with mPTD and preeclampsia remained significant when compared to women with mPTD only. In contrast, the metabolite differences between women with mPTD only and term controls were only seen in phosphatidylethanolamine class. Women with sPTD had significantly different levels of 16 metabolites mainly in amino acid, nucleotide, and steroid classes compared to term controls, of which, anthranilic acid, bilirubin, and steroids also had shared associations in women with mPTD and preeclampsia.

**Conclusion:**

In this sample of US high-risk women, PTD/preeclampsia subgroups each showed some unique and shared associations with maternal postpartum plasma metabolites, including those known to be predictors of future CVD. These findings, if validated, may provide new insight into metabolomic alterations underlying clinically observed PTD/preeclampsia subgroups and implications for women’s future cardiometabolic health.

## Background

Pregnancy is believed to be a vulnerable time period for women’s long-term health. While ample evidence suggests that preterm delivery (PTD) and other pregnancy complications such as preeclampsia are important risk factors for the health of the offspring, much less appreciated is that these same traits may also affect the long-term health of the mother. Pregnancy complications and PTD are associated with risk of having the same conditions in subsequent pregnancies, but also lead to an increased long-term risk of adverse health outcomes, including cardiovascular diseases (CVDs) [1–4], the leading causes of death for women in the USA [5].

All-cause PTDs are associated with a 1.5- to 3-fold higher risk of CVD [1–4, 6]. Previous studies have shown that much of this association is accounted for by metabolic disorders during pregnancy, especially preeclampsia (which explains about 24-26% of the association) [1], or by metabolic disorders postpartum (which may explain about 13-15% of the association) [3]; however, a substantial portion of the risk remains unaccounted for. The PTD-CVD associations persist even in pregnancies uncomplicated by preeclampsia or hypertensive disorders [3, 6, 7], indicating the need to explore other pathways via which PTD and CVD are linked. PTD is heterogeneous, with a variety of different PTD subtypes (i.e., spontaneous vs. medically indicated PTD), with and without preeclampsia, which may affect CVD risk through different pathways. However, such data are quite limited.

We hypothesized that women who experienced different PTD subtypes with and without preeclampsia may exhibit CVD risk factors or biomarkers early in the postpartum period, long before the development of CVD. The identification of such early biomarkers may provide new insight into the biological underpinning of the link between PTD/preeclampsia and women’s future cardiometabolic health and provide targets for prevention and intervention in high-risk women. Metabolomic profiling, via the high-throughput assessment of a large number of circulating small molecule metabolites across multiple pathways, may offer advantages in elucidating biological pathways and biomarker discovery. Recent metabolomic studies have successfully identified multiple metabolites of different classes as early biomarkers for CVD, including branched-chain and aromatic amino acids (i.e., leucine, isoleucine, valine, phenylalanine, and tyrosine) [8–10], short- and medium-chain acylcarnitines [11–13], and metabolites of lipid classes [14–17]. Little is known about whether such metabolites vary in women with PTD vs. term delivery with or without preeclampsia.

In this study, by applying state-of-the-art metabolomic approaches, we quantified maternal metabolomic profiles in postpartum plasma (collected within 1–3 days after delivery) of 1411 women enrolled from the Boston Birth Cohort (BBC), an inner-city multi-ethnic longitudinal study cohort. We aimed to identify both shared and divergent metabolomic patterns (especially in those metabolites associated with CVD risk) for different PTD subtypes with and without preeclampsia, and further to test whether such PTD/preeclampsia-associated metabolomic patterns may vary by maternal characteristics such as ethnicity and parity.

## Methods

### Study population

The study population was a subset of the BBC, an ongoing longitudinal cohort study begun in 1998 at the Boston Medical Center in Massachusetts. Detailed information about the BBC has been reported elsewhere [18, 19]. Briefly, mothers who delivered singleton live births were eligible for the study and were invited to participate within 1 to 3 days after delivery. We excluded pregnancies that were a result of in vitro fertilization, multiple gestations, those with fetal chromosomal abnormalities or major birth defects, and preterm deliveries due to non-obstetric factors (such as trauma). The BBC is a low-income patient population, with a relatively high proportion of PTD and preeclampsia. After obtaining written informed consent, each mother was interviewed using a standardized questionnaire to gather dietary and epidemiologic data, and their electronic medical records (EMRs) were abstracted. Maternal blood samples were obtained within 24–72 h after delivery. The study protocol was approved by the Institutional Review Boards of Boston University Medical Center and the Johns Hopkins Bloomberg School of Public Health.

### Definition of preterm delivery and preeclampsia

Gestational age was assessed by early prenatal ultrasound (< 20 weeks) or based on the first day of the last menstrual period as recorded in maternal EMRs if early prenatal ultrasound was not available [19]. Medically indicated PTD (mPTD) was defined as a delivery by medical induction or Cesarean section (c-section) at < 37 weeks without uterine contractions or rupture of membranes. The indications for medical induction or c-section included gestational complication (mainly preeclampsia), placenta abruption, placenta previa, oligohydramnios, previous c-section, intrauterine growth restriction, absent end diastolic flow, not reassuring fetal heartbeat, fetal distress, signs of reduced amniotic fluid index, or persistent fetal tachycardia*.* Spontaneous PTD (sPTD) was defined as a delivery occurring secondary to documented active preterm labor (uterine contractions with cervical effacement and dilation at < 37 weeks) or premature rupture of membranes at < 37 weeks without uterine contractions or both. We further divided sPTD as early sPTD (spontaneous delivery at < 33 weeks) or late sPTD (spontaneous delivery at 33–36^6/7^ weeks). Physician-diagnosed preeclampsia, eclampsia, and hemolysis, elevated liver enzymes, and low platelet syndromes (HELLP) were extracted from the maternal EMRs and were further confirmed by review of all relevant medical records to meet the definition of preeclampsia according to the recent American College of Obstetricians and Gynecologist (ACOG) criteria [20].

We classified all participating women into one of the following PTD/preeclampsia subgroups: (1) women of term delivery without preeclampsia (including physician-diagnosed preeclampsia, eclampsia, and HELLP), as term controls; (2) women with both mPTD and preeclampsia; (3) women with mPTD but no preeclampsia (referred to as “mPTD only” hereafter); and (4) women with sPTD but no preeclampsia (referred to as “sPTD” hereafter, and further subdivided by early or late sPTD in supplemental analyses). Among the 1411 women with available metabolomic profiles, 9 were removed due to missing preeclampsia status and 72 (61 preeclamptic women with term delivery and 11 preeclamptic women with sPTD) were removed as they did not meet the criteria for either the control or the PTD subgroups as described above.

### Epidemiological and clinical data

Using a standard maternal questionnaire interview [18], maternal epidemiological factors were collected including race/ethnicity, maternal age at delivery, highest education level, parity, smoking during pregnancy, alcohol drinking during pregnancy, illicit drug use during pregnancy, lifetime stress, previous history of PTD, and maternal birthplace (US-born/non-US-born). Maternal pregestational body mass index (BMI), calculated as self-reported weight (kg) divided by height squared (m^2^), was categorized into four groups: normal weight (< 25.0 kg/m^2^), overweight (25–29.9 kg/m^2^), obesity (≥ 30 kg/m^2^), and unknown. Clinical complication before pregnancy, including chronic hypertension and pregestational diabetes, was defined based on archived EMRs.

### Metabolomic profiles and data processing steps

Maternal plasma samples, randomly distributed in each plate, were shipped to the Broad Institute of MIT and Harvard (Cambridge, MA, USA) for metabolomic profiling. Details of this technique can be found elsewhere [21, 22]. Briefly, two liquid chromatography tandem mass spectrometry techniques were applied, including hydrophilic interaction liquid chromatography (HILIC) analyses of water-soluble metabolites in the positive ionization mode and C8 chromatography with positive ion mode analyses of polar and non-polar plasma lipids. Metabolites in different lipid classes, including cholesterol esters (CEs), diacylglycerols (DAGs), triacylglycerols (TAGs), lysophosphatidylcholines (LPCs), lysophosphatidylethanolamines (LPEs), phosphatidylcholines (PCs), and phosphatidylethanolamines (PEs), were further classified based on the number of total acyl chain carbon atoms and double bond contents and were annotated as “C[number of total acyl chain carbon atoms]:[number of double bonds in fatty acid meoeties] [lipid class],” accordingly.

A pooled plasma study reference sample, comprised from all of the studied plasma samples, was evenly distributed throughout the study samples (every ~ 20 study samples) as a quality control step. Using these reference samples, a coefficient of variance (CV) was calculated for each metabolite. Internal standard peak areas were monitored for quality control and to ensure system performance throughout the analyses. A total of 432 known metabolites were successfully quantified, and 40 metabolites with CV > 20%, 10 metabolites unquantifiable in > 10% participants, and 2 xenobiotics were removed from downstream analyses. The remaining 380 metabolites, with any non-detectable values being imputed as one half of the minimal value, were inverse normally transformed to render the distributions approximately Gaussian and to remove the impact of outliers.

### Data analyses

Maternal characteristics were compared between each of the three PTD/preeclampsia subgroups (as defined above) and term controls, using ANOVA tests for continuous variables and *χ*^2^ tests (or Fisher’s exact tests, when necessary) for categorical variables. To identify whether metabolites varied significantly in each PTD/preeclampsia subgroup compared to term controls, we fit a linear regression model with each metabolite as the outcome and the four-categorical index variable (term controls/mPTD and preeclampsia/mPTD only/sPTD) as the exposure, adjusting for conventional and clinical covariates including maternal age at delivery, maternal ethnicity/race, maternal birthplace (US-born versus non-US-born), pregestational BMI category, pregestational diabetes, chronic hypertension, marital status, highest education level, parity, maternal smoking during pregnancy, illicit drug use, lifetime stress, and fetal sex. Bonferroni correction was used to account for multiple testing.

We then performed weighted gene coexpression network analysis (WGCNA) using the *WGCNA* package in R [23], to reduce the number of comparisons made and minimize issues of metabolite collinearity. This method utilizes an unsupervised network-based approach to group metabolites into “modules” based on their correlation patterns. After hierarchical clustering, highly interconnected metabolites were assigned to the same module and was annotated as a unique color. The remaining metabolites (*n* = 51) that were not assigned to any of the established modules were removed from the subsequent analyses. For each module, the top hub metabolite was identified, which was the metabolite having the highest connectivity with other metabolites in the same module. Each participant was then assigned a “score” for each module, which was calculated as the first principal component of the metabolites within the module. The score for each module was then analyzed as the outcome in the subsequent analyses, using the linear regression models with the adjustment of covariates as described above. All analyses were performed using R Software, version 3.6.1.

## Results

### Population characteristics of the study participants

This study included 980 women with term delivery (or term controls) and 350 women with any of the following three PTD/preeclampsia subgroups: (1) mPTD and preeclampsia (*n* = 79), (2) mPTD only (*n* = 52), and (3) sPTD (*n* = 219). The population characteristics across the four study groups are summarized in Table 1. Compared to term controls, women with both mPTD and preeclampsia were older and were more likely to be obese before pregnancy, be stressed, and have chronic hypertension and pregestational diabetes, while women with sPTD were more likely to be unmarried, to be US-born, to smoke during pregnancy, and to have illicit drug use than term controls (all *P* < 0.05). As expected, c-section was significantly more common in women with mPTD, either with (65.8%) or without preeclampsia (80.8%) than in term controls (31.8%).

**Table 1:** Population characteristics of the 1330 women enrolled in the Boston Birth Cohort, stratified by preterm delivery subgroups

Variables	Term delivery	Preterm delivery (PTD) subgroups^**a**^		
		mPTD + preeclampsia	mPTD only	sPTD
*N*	980	79	52	219
Maternal age (years), M ± SD	28.1 ± 6.6	31.1 ± 6.5**	30.2 ± 7.2	28.6 ± 6.4
Ethnicity				
African American	621 (63.4)	61 (77.2)	27 (51.9)	148 (67.6)
White	40 (4.1)	2 (2.5)	2 (3.9)	12 (5.5)
Hispanic	200 (20.4)	12 (15.2)	17 (32.7)	45 (20.5)
Others	119 (12.1)	4 (5.1)	6 (11.5)	14 (6.4)
Maternal birthplace: non-US-born	620 (63.3)	50 (63.3)	33 (63.5)	115 (52.5)**
Married	338 (34.5)	26 (32.9)	17 (32.7)	54 (24.7)*
Pregestational BMI (kg/m^2^) category				
Normal	470 (48.0)	21 (26.6)**	16 (30.8)	104 (47.5)
Overweight	258 (26.3)	24 (30.4)	17 (32.7)	62 (28.3)
Obese	208 (21.2)	32 (40.5)	16 (30.8)	43 (19.6)
Unknown	44 (4.5)	2 (2.5)	3 (5.8)	10 (4.6)
Highest education level				
< High school	273 (27.9)	12 (15.2)	21 (40.4)	62 (28.3)
High school	362 (36.9)	39 (49.4)	18 (34.6)	86 (39.3)
College or above	340 (34.7)	28 (35.4)	13 (25.0)	70 (32.0)
Missing	5 (0.5)	0 (0.0)	0 (0.0)	1 (0.5)
Nulliparity	430 (43.9)	34 (43.0)	17 (32.7)	97 (44.3)
Maternal smoking during pregnancy				
Never	840 (85.7)	62 (78.5)	41 (78.8)	161 (73.5)***
Quitter	64 (6.5)	8 (10.1)	3 (5.8)	20 (9.1)
Current	64 (6.5)	8 (10.1)	8 (15.4)	38 (17.4)
Unknown	12 (1.2)	1 (1.3)	0 (0.0)	0 (0.0)
Alcohol drinking during pregnancy	73 (7.4)	3 (3.8)	5 (9.6)	20 (9.1)
Stress during lifetime				
Mild	410 (41.8)	29 (36.7)*	16 (30.8)	76 (34.7)
Average	490 (50.0)	36 (45.6)	30 (57.7)	118 (53.9)
Stressful	80 (8.2)	14 (17.7)	6 (11.5)	25 (11.4)
Chronic hypertension	37 (3.8)	28 (35.9)***	6 (11.5)*	12 (5.5)
Gestational diabetes	69 (7.0)	9 (11.4)	4 (7.7)	16 (7.3)
Pregestational diabetes	37 (3.8)	12 (15.2)***	4 (7.7)	12 (5.5)
Illicit drug use during pregnancy	66 (6.7)	9 (11.4)	6 (11.5)	34 (15.5)***
Mode of delivery, c-section	311 (31.8)	52 (65.8)***	42 (80.8)***	65 (29.7)
Infant’s sex, male	497 (50.7)	30 (38.0)*	31 (59.6)	123 (56.2)

^a^*mPTD*, medically indicated PTD; the “mPTD + preeclampsia” group refers to women with mPTD and preeclampsia (including preeclampsia, eclampsia, and hemolysis, elevated liver enzymes, and low platelet syndrome (HELLP)); *sPTD*, spontaneous preterm delivery

*P < 0.05; **P < 0.01; ***P < 0.001 for the difference of population characteristics in women of different PTD subgroups compared to term controls, via ANOVA tests and chi-squared tests for continuous and categorical variables, respectively

### Altered plasma metabolites in relation to different PTD/preeclampsia subgroups

**Fig. 1:**
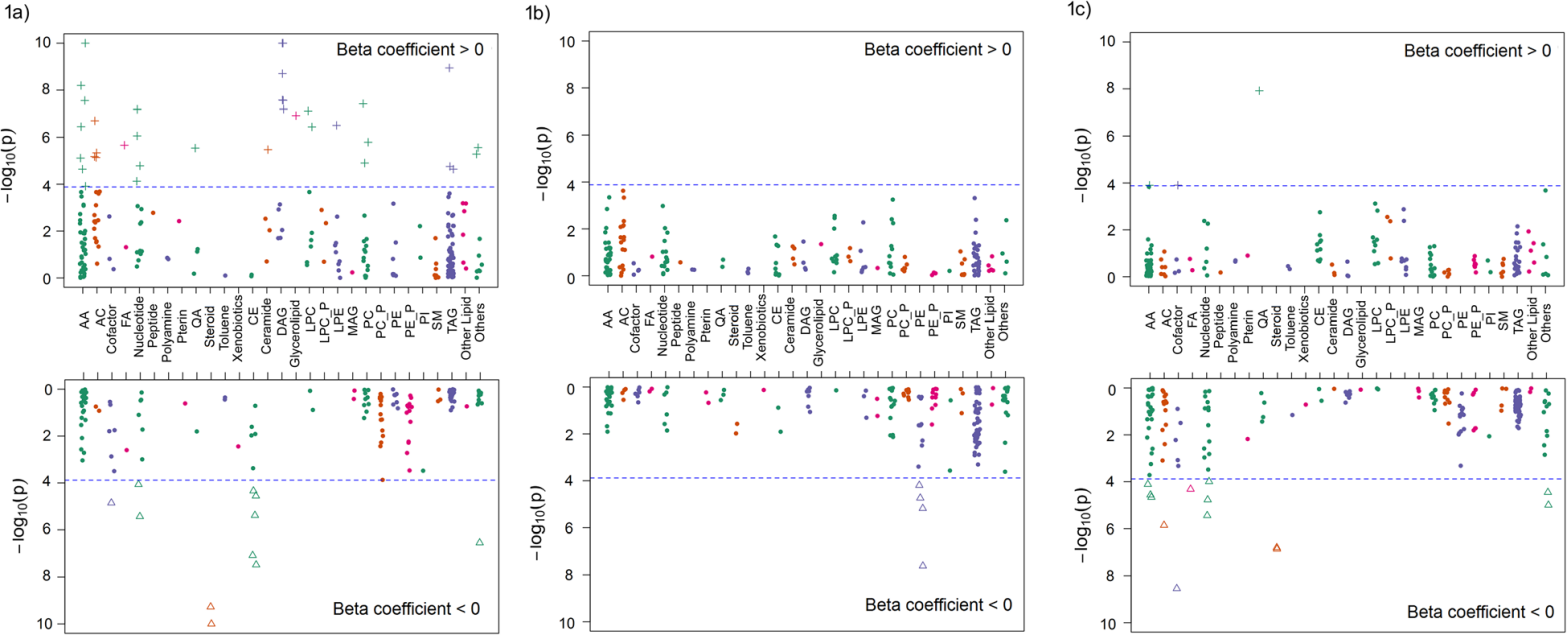
Manhattan plot for the metabolomic differences in women with each preterm delivery (PTD) subgroup compared to women with term delivery. The upper panel presents metabolites that were higher, and the lower panel presents metabolites that were lower in women with medically indicated PTD and preeclampsia (**a**), in women with medically indicated PTD only (**b**), and in women with spontaneous PTD (**c**), respectively. The analyses were adjusted for maternal age at delivery, maternal ethnicity/race, maternal birthplace, pregestational BMI category, pregestational diabetes, chronic hypertension, marital status, highest education level, parity, smoking during pregnancy, illicit drug use, lifetime stress, and fetal sex. *X-axis* represents the metabolite classes. *AA* amino acid, *AC* acylcarnitine, *FA* fatty acid and conjugate, *QA* quaternary amine, *CE* cholesterol ester, *DAG* diacylglycerol, *LPC* lysophosphatidylcholine, *LPC_P* lysophosphatidylcholine plasmalogen, *LPE* lysophosphatidylethanolamine, *MAG* monoacylglycerol, *PC* phosphatidylcholine, *PC_P* phosphatidylcholine plasmalogen, *PE* phosphatidylethanolamine, *PE_P* phosphatidylethanolamine plasmalogen, *PI* phosphatidylinositol, *SM* sphingomyelin, *TAG* triacylglycerol

Figure 1 and Table 2 present the metabolites that were significantly different from term controls in at least one PTD/preeclampsia subgroup. After Bonferroni correction for multiple testing (*P* < 1.3 × 10^−4^) and with term controls as the reference, women with mPTD and preeclampsia had significantly different patterns in 47 metabolites, including higher levels of 36 metabolites that were mainly amino acids (*n* = 7), total carnitine or acylcarnitines (C2, C3, C5-DC, C8, *n* = 4), nucleotides (*n* = 4), DAGs (C34:1, C34:1 DAG/TAG fragment, C34:2, C36:2, C36:3, C38:4, and C38:5, *n* = 7), TAGs (C50:2, C52:2, C52:3, *n* = 3), LPCs (C22:5 and C22:6, *n* = 2), and PCs (C38:6, C40:6, and C40:9, *n* = 3) and lower levels of 11 metabolites including CEs (C14:0, C16:0, C18:0, C18:2, and C18:3, *n* = 5), anthranilic acid, bilirubin, cytosine, 1-methylguanine, and 2 steroids (Table 2 and Additional file 1: Table S1). However, metabolomic differences were modest in women with mPTD only, who had lower levels of 4 PEs (C32:1, C34:2, C36:2, and C36:4) than term controls. In comparison, women with sPTD had significantly lower plasma levels of 13 metabolites (3 amino acids, 3 nucleotides, 2 steroids, C9 carnitine, anthranilic acid, bilirubin, 4-acetamidobutanoate, and trimethylamine-*N*-oxide) and higher levels of 3 metabolites (pyroglutamic acid, niacinamide, and phosphocholine), compared to the term controls. No significant associations were observed between sPTD and lipid metabolites. The identified associations remained largely unchanged when we further adjusted for the timing of maternal blood collection (postpartum day 1, 2, or 3 and above) in the regression models (data not shown).

**Table 2:** Maternal metabolites that were significantly different in women of each preterm delivery (PTD) subgroups, compared to women with term delivery

Maternal metabolites^**a**^	Metabolite classes	mPTD + preeclampsia^**b**^		mPTD only		sPTD	
		Beta ± SE^c^	***P***	Beta ± SE^c^	***P***	Beta ± SE^c^	***P***
Alloisoleucine	Amino acid	− 0.02 ± 0.12	0.88	− 0.32 ± 0.14	0.02	− 0.31 ± 0.07	2.2 × 10^−5^
Creatine	Amino acid	0.98 ± 0.12	1.4 × 10^−15^	0.34 ± 0.14	0.01	0.28 ± 0.07	1.5 × 10^−4^
Creatinine	Amino acid	0.27 ± 0.12	0.02	− 0.03 ± 0.14	0.82	− 0.29 ± 0.07	7.8 × 10^− 5^
Cystine	Amino acid	0.70 ± 0.12	6.4 × 10^−9^	0.08 ± 0.14	0.56	0.02 ± 0.07	0.82
Glutamine	Amino acid	0.52 ± 0.12	2.3 × 10^−5^	0.21 ± 0.14	0.13	0.01 ± 0.07	0.88
Homocitrulline	Amino acid	0.47 ± 0.12	1.2 × 10^−4^	− 0.03 ± 0.14	0.84	− 0.09 ± 0.07	0.22
*N*-Acetyltryptophan	Amino acid	0.68 ± 0.12	2.8 × 10^−8^	− 0.10 ± 0.14	0.47	− 0.10 ± 0.07	0.19
*N*-Acetylputrescine	Amino acid	0.55 ± 0.12	7.7 × 10^−6^	0.22 ± 0.14	0.12	− 0.13 ± 0.07	0.07
N6,N6,N6-trimethyllysine	Amino acid	0.62 ± 0.12	3.5 × 10^−7^	0.29 ± 0.14	0.04	0.08 ± 0.07	0.31
Pyroglutamic acid	Amino acid	0.31 ± 0.12	0.01	0.18 ± 0.14	0.22	0.29 ± 0.08	1.3 × 10^−4^
C3 carnitine	Acylcarnitine	0.57 ± 0.12	4.6 × 10^−6^	0.09 ± 0.14	0.54	0.13 ± 0.08	0.08
C5-DC carnitine	Acylcarnitine	0.63 ± 0.12	2.0 × 10^−7^	0.13 ± 0.14	0.37	− 0.09 ± 0.07	0.21
C8 carnitine	Acylcarnitine	0.54 ± 0.12	7.3 × 10^−6^	0.30 ± 0.14	0.03	− 0.05 ± 0.07	0.52
C9 carnitine	Acylcarnitine	− 0.16 ± 0.12	0.18	− 0.15 ± 0.14	0.28	− 0.36 ± 0.07	1.4 × 10^−6^
Anthranilic acid	Aminobenzoic acid	− 0.63 ± 0.12	2.9 × 10^−7^	− 0.52 ± 0.14	2.4 × 10^−4^	− 0.33 ± 0.08	1.0 × 10^−5^
Bilirubin	Cofactors	− 0.52 ± 0.12	1.4 × 10^−5^	0.15 ± 0.14	0.29	− 0.44 ± 0.07	2.9 × 10^−9^
Niacinamide	Cofactors	0.18 ± 0.12	0.15	0.07 ± 0.14	0.60	0.29 ± 0.08	1.2 × 10^−4^
4-Acetamidobutanoate	Fatty acid	0.57 ± 0.12	2.2 × 10^−6^	− 0.03 ± 0.14	0.84	− 0.30 ± 0.07	4.8 × 10^−5^
Imidazole propionate	Imidazole	0.56 ± 0.12	2.8 × 10^−6^	0.39 ± 0.14	0.004	0.27 ± 0.07	2.1 × 10^−4^
Cytosine	Nucleotide	− 0.56 ± 0.12	3.7 × 10^−6^	0.16 ± 0.14	0.25	− 0.21 ± 0.07	0.01
6,8-Dihydroxypurine	Nucleotide	0.60 ± 0.12	8.8 × 10^−7^	0.24 ± 0.14	0.09	0.09 ± 0.07	0.23
1-Methyladenosine	Nucleotide	− 0.04 ± 0.12	0.72	− 0.00 ± 0.14	0.97	− 0.29 ± 0.07	1.0 × 10^−4^
1-Methylguanine	Nucleotide	− 0.48 ± 0.12	8.6 × 10^−5^	0.04 ± 0.14	0.77	− 0.23 ± 0.08	0.003
7-Methylguanine	Nucleotide	− 0.29 ± 0.12	0.02	− 0.10 ± 0.14	0.48	− 0.35 ± 0.07	3.6 × 10^−6^
Urate	Nucleotide	0.65 ± 0.12	6.5 × 10^−8^	0.29 ± 0.14	0.03	− 0.31 ± 0.07	1.7 × 10^−5^
Carnitine	Quaternary amine	0.58 ± 0.12	2.9 × 10^−6^	0.12 ± 0.14	0.42	− 0.09 ± 0.08	0.24
Phosphocholine	Quaternary amine	0.23 ± 0.12	0.06	0.18 ± 0.14	0.21	0.43 ± 0.08	1.21 × 10^−8^
Cortisol	Steroid	− 0.95 ± 0.12	3.1 × 10^−15^	− 0.35 ± 0.14	0.01	− 0.39 ± 0.07	1.61 × 10^−7^
**Lipids**							
C14:0 CE	Cholesteryl ester	− 0.52 ± 0.11	4.2 × 10^−6^	− 0.33 ± 0.13	0.01	− 0.07 ± 0.07	0.28
C18:2 CE	Cholesteryl ester	− 0.66 ± 0.12	3.4 × 10^−8^	0.00 ± 0.14	0.98	0.09 ± 0.07	0.22
C16:0 Ceramide	Ceramide	0.55 ± 0.12	3.4 × 10^−6^	0.26 ± 0.14	0.06	0.07 ± 0.07	0.30
C34:1 DAG/TAG fragment	Diacylglycerol	0.64 ± 0.12	1.2 × 10^−7^	0.28 ± 0.14	0.04	− 0.01 ± 0.07	0.85
C36:2 DAG	Diacylglycerol	0.78 ± 0.12	4.4 × 10^−11^	0.10 ± 0.13	0.47	− 0.05 ± 0.07	0.47
C38:5 DAG	Diacylglycerol	0.79 ± 0.12	4.5 × 10^−11^	0.15 ± 0.14	0.28	0.09 ± 0.07	0.23
C22:6 LPC	LPC	0.65 ± 0.12	7.7 × 10^−8^	0.41 ± 0.14	0.003	0.25 ± 0.07	7.6 × 10^−4^
C22:6 LPE	LPE	0.62 ± 0.12	3.1 × 10^−7^	0.39 ± 0.14	0.01	0.24 ± 0.07	0.001
C40:6 PC	PC	0.65 ± 0.12	3.8 × 10^−8^	0.38 ± 0.14	0.01	0.12 ± 0.07	0.10
C34:2 PE	PE	− 0.06 ± 0.11	0.62	− 0.74 ± 0.13	2.4 × 10^−8^	− 0.25 ± 0.07	4.8 × 10^−4^
C52:2 TAG	Triacylglycerol	0.71 ± 0.12	1.1 × 10^−9^	0.06 ± 0.13	0.63	− 0.15 ± 0.07	0.04
**Others**							
Methylguanidine	Others	0.56 ± 0.12	5.2 × 10^−6^	− 0.17 ± 0.14	0.23	− 0.10 ± 0.07	0.16
Trimethylamine-*N*-oxide	Others	− 0.05 ± 0.12	0.70	0.04 ± 0.14	0.78	− 0.31 ± 0.07	3.5 × 10^−5^

*LPC* lysophosphatidylcholine, *LPE* lysophosphatidylethanolamine, *PC* phosphatidylcholine, *PE* phosphatidylethanolamine

^a^This table includes metabolites significantly different in any of the three PTD subgroups compared to the term controls, after Bonferroni adjustment for multiple testing (*P* < 1.3 × 10^−4^). For two or more highly correlated metabolites (with *r* > 0.60), only one representative metabolite is included in the table, and the others are shown in Supplementary Table S1

^b^Including preeclampsia, eclampsia, and hemolysis, elevated liver enzymes, and low platelet syndrome (HELLP)

^c^Adjusted for an array of conventional and clinical factors, including maternal age at delivery, maternal ethnicity/race, maternal birthplace pregestational BMI category, pregestational diabetes, chronic hypertension, marital status, highest education level, parity, smoking during pregnancy, illicit drug use, lifetime stress, and fetal sex

We further examined the 47 metabolites that were associated with the group of mPTD and preeclampsia, compared to women with mPTD only. At *P* < 0.001, women with both mPTD and preeclampsia had higher levels of four DAGs (C34:1, C34:2, C38:4, and C52:2) and two TAGs (C50:2 and C52:2) and lower levels of four CEs (C16:0, C18:0, C18:2, and C18:3) than women with mPTD only, suggesting specific associations with preeclampsia.

### Metabolite patterns related to PTD/preeclampsia subgroups by metabolomic network analyses

**Fig. 2:**
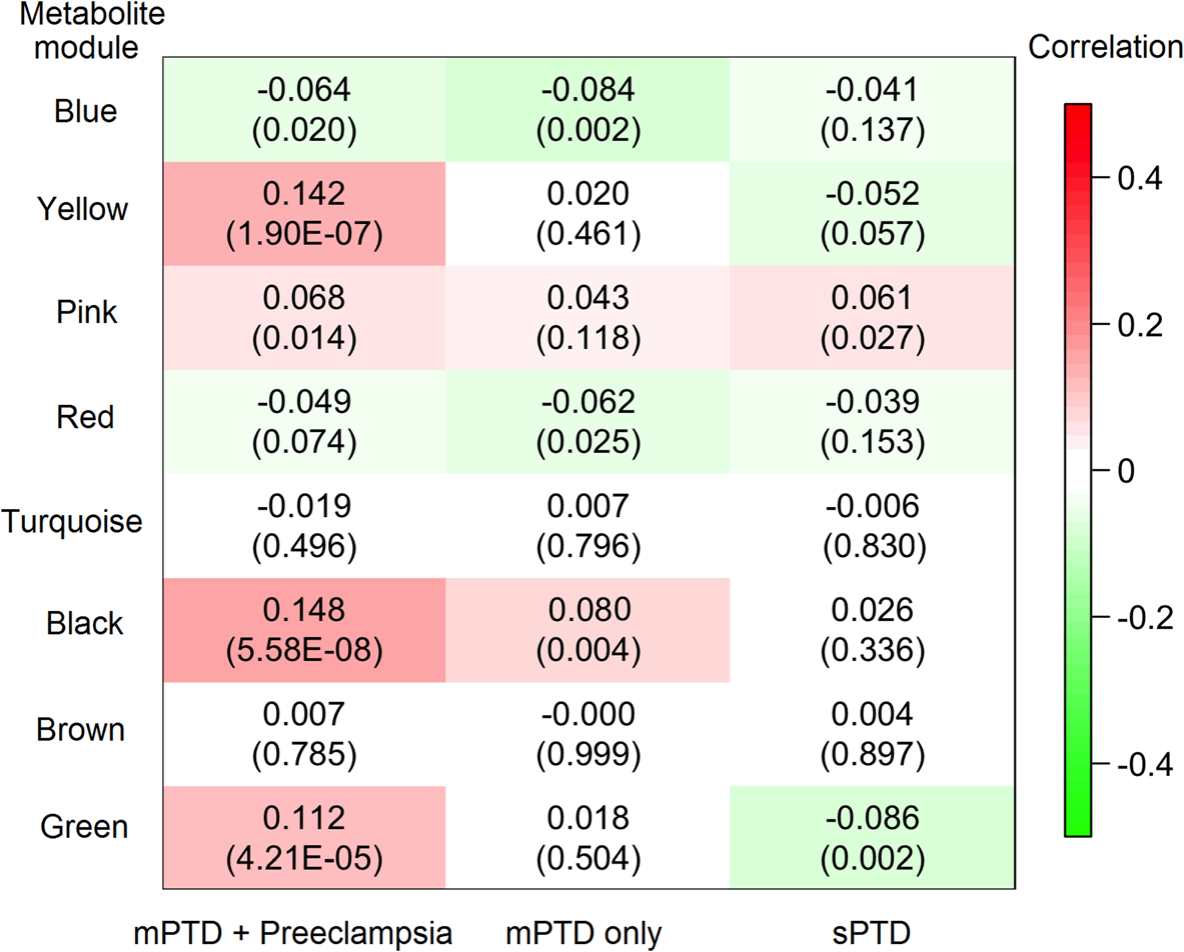
Correlations and *p*-values (in parentheses) of each coexpressed metabolite module with different preterm delivery (PTD) subgroups. The metabolite modules were built via the WGCNA analysis on 1330 women using the “unsigned” network

With WGCNA network analysis, 329 metabolites were grouped into 8 metabolic network modules, annotated as different colors. Metabolites in each module are presented in Additional file 1: Table S2. Figure 2 presents the correlations between each PTD/preeclampsia subgroup and each module score. With the adjustment of covariates and with *P* < 0.006 (= 0.05/8 modules) as the significance cutoff, we observed that women with mPTD and preeclampsia had significantly higher scores for the “yellow” (C16:0 CE as the top hub metabolite that was inversely correlated with the module score, *P* = 7.8 × 10^−7^), “black” (C14:1 carnitine as the top hub metabolite, *P* = 1.6 × 10^−5^), and “green” modules (pseudouridine as the top hub metabolite, *P* = 0.0007) than term controls (Table 3). Women with mPTD only had a significantly lower score of the “blue” module (*P* = 0.004), while women with sPTD had a significantly lower score of the “green” module (*P* = 0.003, Table 3), compared to term controls.

**Table 3:** Associations of the eight metabolite modules with each preterm delivery (PTD) subgroup, compared to women with term delivery

Module^a^	*N*_metabolite_	Top hub metabolite^b^	mPTD + preeclampsia		mPTD only		sPTD	
			Beta ± SE^c^	*P*	Beta ± SE^c^	*P*	Beta ± SE^c^	*P*
Blue	54	C44:1 TAG	0.000 ± 0.003	0.98	− 0.011 ± 0.004	0.004*	− 0.003 ± 0.002	0.10
Yellow	38	C16:0 CE or C36:2 DAG^d^	0.016 ± 0.003	7.8 × 10^–7^*	− 0.001 ± 0.004	0.86	− 0.004 ± 0.002	0.06
Pink	26	C18:1 LPC	0.009 ± 0.003	0.007	0.005 ± 0.004	0.24	0.005 ± 0.002	0.03
Red	36	C32:2 PC	0.001 ± 0.003	0.76	− 0.008 ± 0.004	0.03	− 0.002 ± 0.002	0.30
Turquoise	63	C16:0 sphingomyelin	− 0.001 ± 0.003	0.74	0.001 ± 0.004	0.71	0.000 ± 0.002	0.85
Black	34	C14:1 carnitine	0.015 ± 0.003	1.6 × 10^–5^*	0.009 ± 0.004	0.02	0.002 ± 0.002	0.33
Brown	41	Valine	0.003 ± 0.003	0.38	0.001 ± 0.004	0.77	0.001 ± 0.002	0.66
Green	37	Pseudouridine	0.011 ± 0.003	7.0 × 10^–4^*	0.002 ± 0.004	0.54	− 0.006 ± 0.002	0.003*

*mPTD* medically indicated PTD, *CE* cholesteryl ester, *DAG* diacylglycerol, *LPC* lysophosphatidylcholine, *PC* phosphatidylcholine, *TAG* triacylglycerol

^a^The WGCNA analysis was conducted on 1330 samples using the “unsigned” network, where the highly correlated (either positive or negative) metabolites were grouped into the same module

^b^The top hub metabolite was defined as the one with the highest mean correlation with the other metabolites in the same module

^c^Adjusted for maternal age at delivery, maternal ethnicity/race, maternal birthplace, pregestational BMI category, pregestational diabetes, chronic hypertension, marital status, highest education level, parity, smoking during pregnancy, illicit drug use, lifetime stress, and fetal sex

^d^C16:0 CE is negatively correlated with the “yellow” module score while C36:2 DAG is positively correlated with the “yellow” module score

*Significant after Bonferroni corrections for multiple testing on 8 metabolite modules

Stratified analyses were further performed by maternal ethnicity and maternal parity. As shown in Additional file 1: Table S3 and Table S4, the associations between the PTD/preeclampsia subgroups and each metabolite module were largely comparable between Black and non-Black mothers and between nulliparity and multiparity mothers.

### Exploratory analyses of early and late sPTD

When women with sPTD were split into early (< 33 weeks of gestation, *n* = 67) and late sPTD (33–36^6/7^ weeks of gestation, *n* = 152), and compared to term controls, we observed that the identified sPTD-associated differences in 16 individual metabolites were predominantly observed in women with early sPTD rather than in women with late sPTD (Additional file 1: Table S5). In addition, another 20 metabolites were significantly different in women with early sPTD, including 6 lipids (4 CEs and 2 LPCs) that were higher and 4 lipids (3 PEs and C36:2 phosphatidylinositol) that were lower in mothers with early sPTD (Additional file 1: Table S5 and Additional file 2: Fig. S1). Of note, the identified two LPCs (C22:5 and C22:6) were also significantly higher in women with mPTD and preeclampsia, and two PEs (C34:2 and C36:2) that were lower in women with early sPTD were also significantly lower in women with mPTD only. In the WGCNA analyses, women with early sPTD had significantly lower scores of the “yellow” (C16:0 CE as the top hub metabolite) and “green” (pseudouridine as the top hub metabolite) modules than term controls (Additional file 1: Table S6).

## Discussion

To our knowledge, this study represents the largest to date to explore postpartum metabolomic profiles significantly associated with PTD/preeclampsia subgroups from a high-risk US multi-ethnic population. A total of 47, 4, and 16 metabolites were significantly different in women with mPTD and preeclampsia, in women with mPTD only, and in women with sPTD, respectively, compared with term controls. The broad-scale metabolite differences in women with mPTD and preeclampsia predominantly involve acylcarnitines and lipids. The neutral lipids, including DAGs, TAGs, and CEs, were significantly higher in women with mPTD and preeclampsia than in women with mPTD only, suggesting potential specific associations with preeclampsia. This finding is in line with clinically observed significant and independent adverse impact of preeclampsia-related PTD on maternal cardiometabolic health [1, 3]. We also observed significant lipid metabolite alteration in mothers with early sPTD, including those lipids (LPCs and PEs) that were associated with medically indicated PTD, indicating shared pathways among different PTD subtypes in affecting maternal metabolic health. While a comprehensive discussion on the differences of all the metabolites identified in this study is beyond the word limit of this report, below we just highlight the major findings and relevant literature.

### Differences in acylcarnitine associated with mPTD and preeclampsia

Significantly higher levels of multiple acylcarnitines (metabolites related to cellular energy production) were mainly observed in women with mPTD and preeclampsia. Acylcarnitines are responsible for the transport of long-chain fatty acids from the cytoplasm into the mitochondria for β-oxidation. Fatty acids play an important role during pregnancy as metabolic fuel for the placenta [24]. Elevated acylcarnitines have been reported as potential biomarkers for preeclampsia [25–27]. Using the network analyses via WGCNA, acylcarnitines together with metabolites in purine metabolism (6,8-dihydroxypurine, xanthine, and xanthosine), which all were higher in women with mPTD and preeclampsia, were classified into the “black” module, suggesting coexpression of these metabolites.

### Alterations in lipid metabolites among PTD/preeclampsia subgroups

Neutral lipids, including DAGs, TAGs, and CEs, constitute the most abundant group of lipids and serve as energy and carbon storage. These lipids, or the score for the “yellow” module, were significantly altered in women with mPTD and preeclampsia compared to women with mPTD only, suggesting preeclampsia-specific associations. Accumulation of DAGs has been reported to play a role in insulin resistance in cell [28]. DAGs are precursors to TAGs which play an important role in the storage of energy and fatty acids. In our study, the major TAGs that were higher in women with mPTD and preeclampsia included TAG C50:2, C52:2, and C52:3. These TAGs are of short- or medium-carbon chains which are likely hydrolyzed to release saturated (i.e., C16:0 palmitic acid or C18:0 stearic acid) and monounsaturated (i.e., C18:1 oleic acid) fatty acids. Oxidation of saturated and monounsaturated fatty acids provides more energy than oxidation of polyunsaturated fatty acids of the same length [29]. Accumulation of such short- or medium-carbon chain TAGs may indicate dysfunctional energy metabolism and may induce pro-inflammatory signaling [30], which has been proposed as a feature of multiple PTD-related diseases, including diabetes [22, 31] and CVD [14]. Another type of neutral lipid, CEs, was inversely correlated with DAGs and TAGs, which may partly explain the lower levels of CEs (C14:0, C16:0, C18:0, C18:2, and C18:3) that we observed in women with mPTD and preeclampsia. Consistently, lower CEs have been linked to unhealthy lifestyles including a lack of physical activity (including C16:0 and C18:2) [32] and an insulinemic dietary pattern (including C14:0 and C18:3) [33]. However, the relationships between CEs and CVD risk have been inconclusive, including both positive [14] and inverse associations [34, 35]. In comparison, we observed that multiple CEs (C18:0, C20:3, C20:4, and C22:4) were positively associated with early sPTD and that the score for the "yellow" module was inversely associated with early sPTD in this study. Future studies are needed to explore whether and how differences in levels of such neutral lipids may explain the adverse impact of preeclampsia-related PTD and early sPTD on CVD risk.

### Altered metabolites of amino acids and nucleotides by different PTD/preeclampsia subgroups

Different levels of amino acids and nucleotides were observed in women with mPTD and preeclampsia and in women with sPTD (especially those with early sPTD), including both shared and unique metabolites. For example, anthranilic acid and bilirubin were lower across the different PTD subtypes. Anthranilic acid is the metabolite of tryptophan. Tryptophan level during pregnancy was lower in mothers with PTD [36]. Anthranilic acid derivatives have been reported to have anti-inflammatory function [37, 38]. Collectively, this evidence may help to explain the inverse relationship between PTD and anthranilic acid. Bilirubin is a bile pigment with potent antioxidant properties that inhibit lipid oxidation and atherosclerotic development [39, 40]. A number of studies have reported an inverse association between bilirubin levels and risk of hypertension, diabetes, and CVD [41, 42]. Taken together, these findings suggest the hypothesis that inflammation and elevated oxidative stress are potential mechanisms via which both preeclampsia-related mPTD and early sPTD may influence future cardiometabolic health.

The main strengths of our study included a large sample size, a well-validated metabolomic platform and detailed information on covariates, and more importantly, our ability to define PTD/preeclampsia subgroups, which are clinically and pathogenically important to gain new insight. Some limitations of our study should also be acknowledged. First, the BBC was particularly designed to study preterm delivery in an inner-city, predominantly minority population; therefore, caution is needed when generalizing our findings to other populations. Second, there is lack of a replication cohort in this study. We cannot exclude the possibility that some of these findings may not be replicated in another cohort. Third, maternal metabolites were measured in plasma collected within 1–3 days after delivery, which was at a time of significant flux with respect to molecules in the circulation. Future studies are needed to investigate whether the identified metabolite differences (especially those associated with mPTD and preeclampsia) are persistent across different time periods postpartum and whether they are associated with future CVD risk. Fourth, we could not distinguish early-onset versus late-onset preeclampsia in this study. Given the possibility that prolonged exposure to preeclampsia may be associated with an increased risk of CVD, further studies are needed to test whether early-onset preeclampsia and late-onset preeclampsia have different associations with maternal postpartum metabolomic profiles. Fifth, pregestational cardiovascular risk factors are important risk factors of gestational complications including preeclampsia [43–45] and they may also lead to metabolomic differences postpartum. To minimize potential confounding effects, we adjusted for pregestational overweight/obesity, hypertension, and diabetes in our analyses. Pregestational dyslipidemia is another potential risk factor of preeclampsia [44, 45]. However, information on pregestational dyslipidemia was not available in this study. Future studies are needed to assess potential confounding by pregestational dyslipidemia. Finally, we were only able to further stratify preterm delivery into early vs. late for the sPTD group, but not for other mPTD/preeclampsia subgroups due to limited sample size.

## Conclusion

We demonstrated individual and clusters of maternal postpartum metabolites that were significantly and specifically associated with clinically observed subgroups: mPTD and preeclampsia, mPTD only, and sPTD, as well as some metabolites that were shared among the subgroups. Some of the identified lipid metabolites and/or related pathways, especially those associated with the mPTD and preeclampsia subgroup, have been reported as early predictors of future risk of CVD. Our study represents an early attempt to better understand metabolomic underpinnings of PTD/preeclampsia. Additional studies are warranted to confirm our findings and further investigate the link of these identified metabolites in mediating or modifying a woman’s future cardiometabolic risk. A better understanding of the pathways from PTD/preeclampsia, postpartum metabolomic profiles, to maternal future cardiometabolic risk may lead to new risk assessment and intervention strategies and help to identify at-risk women early in the disease process when intervention may delay or prevent the onset of cardiometabolic disease.

## Supplementary information

**Additional file 1: Table S1.** Other maternal metabolites that were significantly different in women of each preterm delivery (PTD) subgroup, compared to women of term delivery. **Table S2.** Individual metabolites included in each of the 8 metabolite modules. **Table S3.** Associations of metabolite modules with different preterm delivery (PTD) subgroups, stratified by maternal parity. **Table S4.** Associations of metabolite modules with different preterm delivery (PTD) subgroups, stratified by maternal race / ethnicity. **Table S5.** Maternal metabolites that were significantly different in women with late sPTD and in women with early sPTD, separately, compared to women with term delivery. **Table S6.** Associations of metabolite modules with early and late sPTD, separately, compared to women with term delivery.

**Additional file 2: Figure S1.** Manhattan plot for the metabolomic differences in women with early and late spontaneous PTD, separately, compared to women with term delivery.

## Data Availability

The datasets used and/or analyzed for the current study are available from the corresponding author on reasonable request, and after the Institutional Review Board review and approval.

## Abbreviations

BBC: Boston Birth Cohort
BMI: Body mass index
CE: Cholesteryl ester
CV: Coefficient of variance
CVD: Cardiovascular disease
DAG: Diacylglycerol
EMR: Electronic medical record
LPC: Lysophosphatidylcholine
LPE: Lysophosphatidylethanolamine
mPTD: Medically indicated PTD
PC: Phosphatidylcholine
PE: Phosphatidylethanolamine
PI: Phosphatidylinositol
PTD: Preterm delivery
sPTD: Spontaneous PTD
TAG: Triacylglycerol
WGCNA: Weighted gene coexpression network analysis
