# Supplementary material for: Postpartum plasma metabolomic profile among women with preeclampsia and preterm delivery: implications for long-term health
Source: BMC Med. 2020 Oct 13;18:277. doi: 10.1186/s12916-020-01741-4 (PMC7552364; doi:10.1186/s12916-020-01741-4)
Supplement: Supplementary file 1 — Additional file 1: Table S1. Other maternal metabolites that were significantly different in women of each preterm delivery (PTD) subgroup, compared to women of term delivery. Table S2. Individual metabolites included in each of the 8 metabolite modules. Table S3. Associations of metabolite modules with different preterm delivery (PTD) subgroups, stratified by maternal parity. Table S4. Associations of metabolite modules with different preterm delivery (PTD) subgroups, stratified by maternal race / ethnicity. Table S5. Maternal metabolites that were significantly different in women with late sPTD and in women with early sPTD, separately, compared to women with term delivery. Table S6. Associations of metabolite modules with early and late sPTD, separately, compared to women with term delivery. [file 12916_2020_1741_MOESM1_ESM.docx]

**Table S1**. Other maternal metabolites that were significantly different in women of each preterm delivery (PTD) subgroup, compared to women of term delivery.

| **Metabolite ^a^** | **Class** | **mPTD + Preeclampsia** | |  | **mPTD only** | | |  | | **sPTD** | |  |
| --- | --- | --- | --- | --- | --- | --- | --- | --- | --- | --- | --- | --- |
|  |  | **Beta±Se ^b^** | ***P*** | | | **Beta±Se ^b^** | ***P*** | | **Beta±Se ^b^** | | ***P*** | |
| SDMA | Amino Acid | 0.42±0.12 | 0.0008 | | | -0.16±0.14 | 0.26 | | -0.32±0.08 | | 2.7×10^-5^ | |
| C2 carnitine | Acylcarnitine | 0.55±0.12 | 6.7×10^-6^ | | | 0.38±0.14 | 0.01 | | -0.09±0.07 | | 0.25 | |
| Xanthosine | Nucleotide | 0.48±0.12 | 7.5×10^-5^ | | | 0.20±0.14 | 0.16 | | 0.14±0.07 | | 0.06 | |
| Xanthine | Nucleotide | 0.54±0.12 | 1.6×10^-5^ | | | 0.18±0.14 | 0.20 | | 0.22±0.08 | | 0.004 | |
| Cortisone | Steroid | -0.75±0.12 | 5.4×10^-10^ | | | -0.31±0.14 | 0.03 | | -0.39±0.07 | | 1.4×10^-7^ | |
| C16:0 CE | Cholesteryl ester | -0.49±0.12 | 2.8×10^-5^ | | | 0.09±0.14 | 0.50 | | 0.15±0.07 | | 0.03 | |
| C18:0 CE | Cholesteryl ester | -0.49±0.12 | 4.6×10^-5^ | | | 0.02±0.14 | 0.89 | | 0.23±0.07 | | 0.002 | |
| C18:3 CE | Cholesteryl ester | -0.64±0.12 | 8.4×10^-8^ | | | 0.04±0.14 | 0.75 | | 0.10±0.07 | | 0.18 | |
| C34:1 DAG | Diacylglycerol | 0.65±0.12 | 6.4×10^-8^ | | | -0.04±0.14 | 0.77 | | -0.05±0.07 | | 0.51 | |
| C34:2 DAG | Diacylglycerol | 0.67±0.12 | 2.8×10^-8^ | | | -0.06±0.14 | 0.66 | | -0.03±0.07 | | 0.63 | |
| C36:3 DAG | Diacylglycerol | 0.72±0.12 | 2.0×10^-9^ | | | 0.08±0.14 | 0.54 | | -0.09±0.07 | | 0.24 | |
| C38:4 DAG | Diacylglycerol | 0.66±0.12 | 2.7×10^-8^ | | | -0.01±0.14 | 0.92 | | -0.03±0.07 | | 0.67 | |
| C22:5 LPC | Lysophosphatidylcholine | 0.62±0.12 | 3.6×10^-7^ | | | 0.41±0.14 | 0.003 | | 0.13±0.07 | | 0.08 | |
| C38:6 PC | Phosphatidylcholine | 0.52±0.12 | 1.3×10^-5^ | | | 0.24±0.14 | 0.07 | | 0.07±0.07 | | 0.31 | |
| C40:9 PC | Phosphatidylcholine | 0.55±0.11 | 1.6×10^-6^ | | | 0.38±0.13 | 0.004 | | 0.13±0.07 | | 0.06 | |
| C32:1 PE | Phosphatidylethanolamine | 0.16±0.11 | 0.15 | | | -0.52±0.13 | 6.4×10^-5^ | | -0.17±0.07 | | 0.01 | |
| C36:2 PE | Phosphatidylethanolamine | -0.13±0.12 | 0.28 | | | -0.61±0.13 | 6.6×10^-6^ | | -0.18±0.07 | | 0.01 | |
| C36:4 PE | Phosphatidylethanolamine | 0.04±0.12 | 0.76 | | | -0.60±0.14 | 1.8×10^-5^ | | -0.19±0.07 | | 0.01 | |
| C50:2 TAG | Triacylglycerol | 0.50±0.12 | 2.3×10^-5^ | | | -0.16±0.13 | 0.23 | | -0.10±0.07 | | 0.17 | |
| C52:3 TAG | Triacylglycerol | 0.52±0.12 | 1.8×10^-5^ | | | 0.13±0.14 | 0.36 | | -0.11±0.07 | | 0.13 | |
|  |  |  |  | | |  |  | |  | |  | |

mPTD: medically-indicated PTD; sPTD: spontaneous PTD.

^a^ This table includes Symmetric dimethylarginine (SDMA, highly correlated with creatine); C2 carnitine (highly correlated with C14:1 carnitine); xanthine and xanthosine (highly correlated with 6,8-dihydroxypurine); Cortisone (highly correlated with cortisol); C16:0 and C18:0 CE (highly correlated with C18:2 CE), C18:3 CE (highly correlated with C14:0 CE); C34:1, C34:2, C36:3 and C38:4 DAG (all highly correlated with C36:2 DAG); C22:5 LPC (highly correlated with C22:6 LPC); C38:6 and C40:9 PC (highly correlated with C40:6 PC); C32:1, C36:2 and C36:4 PE (highly correlated with C34:2 PE); as well as C50:2 and C52:3 TAG (highly correlated with C52:2 TAG).

^b^ Adjusted for an array of conventional and clinical factors, including maternal age at delivery, maternal ethnicity/race, maternal birthplace (US-born vs non-US-born), maternal pregestational BMI category, pregestational diabetes, chronic hypertension, marital status, highest education level, parity, smoking during pregnancy, illicit drug use, lifetime stress, and fetal sex.

| **Table S2.** Individual metabolites included in each of the 8 metabolite modules. | | |
| --- | --- | --- |
| **Module** | **N** | **Metabolites ^a,b,c^ included in the modules ^d^** |
| Black | 34 | Carnitines (C2^a^, C4-OH, C6, C7, C8^a^, C10, C12, C12:1, C14, C14:1, C14:2, C16, C18:1, C18:2), 6,8-dihydroxypurine^a^, aminoisobutyric acid, diacetylspermine, glutamate, hypotaurine, hypoxanthine, inosine, linoleoyl ethanolamide, N-oleoylethanolamine, N1,N12-diacetylspermine, niacinamide^c^, palmithoylethanolamide, phosphocholine^c^, pyroglutamic acid^c^, sphinganine, sphingosine, taurine, threo-sphingosine, xanthine^a^, xanthosine^a^ |
| Blue | 54 | C34:0 DAG, TAG (C42:0, C43:0, C43:1, C43:2, C44:0, C44:1, C44:2, C45:0, C45:1, C45:2, C45:3, C46:0, C46:1, C46:2, C46:3, C46:4, C47:0, C47:1, C47:2, C48:0, C48:1, C48:2, C48:3, C48:4, C48:5, C49:0, C49:2, C49:3, C50:0, C50:1, C51:0, C51:1, C51:2, C52:0, C52:1, C54:1, C54:6, C55:2, C56:1, C56:2, C56:5, C56:6, C56:7, C56:8, C56:9, C58:10, C58:11, C58:7, C58:8, C58:9, C60:12), thiamine, trigonelline |
| Brown | 41 | acetylglutamic acid, ADMA, alanine, alpha-glutamyllysine, arginine, asparagine, beta-alanine, betaine, ACs (C3^a^, C4, C5), choline, citrulline, creatine^a^, GABA, glutamine^a^, glycine, guanidinoacetic acid, histidine, homoarginine, hydroxyectoine, hydroxyproline, isoleucine, leucine, lysine, methionine, methionine sulfoxide, N-acetylhistidine, N-alpha-acetylarginine, NMMA, ornithine, phenylalanine, pipecolic acid, proline, pyridoxamine, sarcosine, serine, threonine, tryptophan, tyrosine, valine |
| Green | 37 | 1-methylguanine^a^, 1-methylguanosine, 3-methylhistidine, 4-acetamidobutanoate^a,c^, 5-hydroxymethyl-4-methyluracil, 7-methylguanine^c^, acetyl-galactosamine, butyrobetaine, ACs (C3-DC-CH3, C5-DC^a^, C5:1, C9^c^), carnitine^a^, creatinine^c^, dimethylglycine, homocitrulline^a^, isoxanthopterin, kynurenic acid, methylguanidine^a^, methylimidazoleacetic acid, N-acetyalanine, N-acetylaspartic acid, N-acetylgalactosamine, N-acetylornithine, N-acetyltryptophan^a^, N-carbamoyl-beta-alanine, N1-acetylspermidine, N2,N2-dimethylguanosine, N4-acetylcytidine, N6-acetyllysine, N6,N6-dimethyllysine, N6,N6,N6-trimethyllysine^a^, pantothenate, pseudouridine, ribothymidine, SDMA^c^, urate ^a,c^ |
| Pink | 26 | LPC (C16:0, C16:1, C18:0, C18:1, C18:2, C18:3, C20:4, C20:5, C22:5^a^, C22:6^a^); LPE (C16:0, C18:0, C18:1, C18:2, C20:0, C20:1, C20:4, C22:0, C22:6^a^); LPC plasmalogen (C16:1, C18:1, C18:1 plasmalogen_minor); C16:1 MAG, C36:4 hydroxy-PC, 1-methyladenosine^c^, alpha-glycerophosphocholine |
| Red | 36 | C14:0 LPC, C16:1 CE, PC (C30:0, C30:1, C32:1, C32:2, C34:1, C34:2, C34:2 PC-B, C34:3, C34:4, C36:1, C36:2, C36:2 PC-B, C36:3, C36:4 PC-A, C38:2,C38:3), PE (C32:0, C32:1^b^, C34:0, C34:2^b^, C36:0, C36:1, C36:2^b^, C36:3, C36:4^b^, C38:4, C38:5, C38:6, C40:6), PI (C36:2, C36:4), TAG (C50:5, C50:6, C52:7) |
| Turquoise | 63 | 1,2,4-trimethylbenzene, C26 carnitine, gabapentin, thyroxine, trimethylbenzene, Ceramide (C16:0^a^,C22:0, C24:0, C24:1), SM (C14:0, C16:0, C16:1, C18:0, C18:1, C18:2, C20:0, C22:0, C24:0, C24:1), PC (C32:0, C34:0, C36:0, C36:4 PC-B, C38:4, C38:6^a^, C40:10, C40:6^a^, C40:9^a^), PC plasmalogen (C34:1-A, C34:1-B, C34:2, C34:3, C34:5, C36:1, C36:2, C36:3, C36:4, C36:5 -A, C36:5 -B, C38:4, C38:6, C38:7, C40:7), PE plasmalogen (C34:2, C34:3, C36:1, C36:2, C36:3, C36:4, 36:5, C38:3, C38:5, C38:6, C38:7, C40:7) , C34:0 PS, PS plasmalogen (C36:2, C36:3), C38:2 PE, C38:4 PI, C54:10 TAG, campesterol, cholesterol |
| Yellow | 38 | CE (C14:0^a^, C16:0^a^, C18:0^a^, C18:1, C18:2^a^, C18:3^a^, C20:3, C20:4, C20:5, C22:4, C22:6); DAG (C32:0, C32:1, C34:1^a^, C34:1 DAG_or_TAG^a^, C34:2^a^, C36:1, C36:2^a^, C36:3^a^, C36:4, C38:4^a^); TAG (C50:2^a^, C50:3, C50:4, C51:3, C52:2^a^, C52:3^a^, C52:4, C52:5, C53:2, C53:3, C54:2, C54:3, C54:4, C54:5, C55:3, C56:3, C56:4) |

**^a,b,c^** The individual metabolite was significantly different in ^a^ women with mPTD and preeclampsia, ^b^ women with mPTD only, and ^c^ women with sPTD, respectively, compared to women of term delivery.

^d^ The metabolite modules were built via the WGCNA analysis on 1,330 women using the ’unsigned’ network.

**Table S3.** Associations of metabolite modules with different preterm delivery (PTD) subgroups, stratified by maternal parity.

| Metabolite module | mPTD + preeclampsia | | | mPTD only | | | sPTD | | |
| --- | --- | --- | --- | --- | --- | --- | --- | --- | --- |
|  | Beta±SE ^a^ | *P* |  | Beta±SE^a^ | *P* |  | Beta±SE ^a^ | *P* | |
| **Nulliparity** (n=430 term controls; 34 with mPTD and preeclampsia; 17 with mPTD only and 97 with sPTD) | | | | | | | | |  |
| Blue | 0.000±0.005 | 0.93 | | -0.003±0.006 | 0.61 | | 0.001±0.003 | 0.78 | |
| Yellow | 0.020±0.005 | 3.6×10^-5^ | | -0.005±0.006 | 0.47 | | -0.002±0.003 | 0.52 | |
| Pink | 0.008±0.005 | 0.12 | | 0.005±0.007 | 0.51 | | 0.008±0.003 | 0.01 | |
| Red | 0.002±0.005 | 0.69 | | -0.009±0.006 | 0.18 | | 0.002±0.003 | 0.59 | |
| Turquoise | 0.000±0.005 | 0.94 | | 0.000±0.007 | 0.96 | | 0.002±0.003 | 0.45 | |
| Black | 0.023±0.005 | 6.8×10^-6^ | | -0.000±0.007 | 0.98 | | 0.000±0.003 | 0.99 | |
| Brown | 0.009±0.005 | 0.08 | | 0.007±0.007 | 0.28 | | 0.004±0.003 | 0.25 | |
| Green | 0.018±0.005 | 8.5×10^-4^ | | 0.002±0.007 | 0.75 | | -0.008±0.003 | 0.01 | |
| **Multiparity** (n=550 term controls; 45 with mPTD and preeclampsia; 35 with mPTD only; and 122 with sPTD) | | | | | | | | |  |
| Blue | 0.001±0.004 | 0.87 | | -0.015±0.005 | 0.002 | | -0.006±0.003 | 0.03 | |
| Yellow | 0.014±0.005 | 0.002 | | 0.000±0.005 | 0.93 | | -0.005±0.003 | 0.10 | |
| Pink | 0.009±0.005 | 0.04 | | 0.005±0.005 | 0.30 | | 0.002±0.003 | 0.52 | |
| Red | 0.000±0.005 | 0.97 | | -0.009±0.005 | 0.06 | | -0.005±0.003 | 0.08 | |
| Turquoise | -0.003±0.005 | 0.50 | | 0.001±0.005 | 0.77 | | -0.001±0.003 | 0.61 | |
| Black | 0.009±0.005 | 0.05 | | 0.015±0.005 | 0.002 | | 0.003±0.003 | 0.27 | |
| Brown | -0.001±0.005 | 0.87 | | -0.001±0.005 | 0.87 | | -0.001±0.003 | 0.72 | |
| Green | 0.007±0.004 | 0.10 | | 0.003±0.005 | 0.53 | | -0.005±0.003 | 0.06 | |

^a^ Compared to women with term delivery, with the adjustment of an array of conventional and clinical factors, including maternal age at delivery, maternal ethnicity/race, maternal birthplace (US-born vs non-US-born), maternal pregestational BMI category, pregestational diabetes, chronic hypertension, marital status, highest education level, smoking during pregnancy, illicit drug use, lifetime stress, and fetal sex.

**Table S4.** Associations of metabolite modules with different preterm delivery (PTD) subgroups, stratified by maternal race / ethnicity.

| Metabolite Module | mPTD + Preeclampsia ^a^ | | mPTD only ^a^ | | sPTD ^a^ | |
| --- | --- | --- | --- | --- | --- | --- |
|  | Beta±SE | *P* | Beta±SE | *P* | Beta±SE | *P* |
| **Black** (n= 621 term controls; 61 with mPTD and preeclampsia; 27 with mPTD only; and 148 with sPTD) | | | | | | |
| Blue | 0.001±0.004 | 0.68 | -0.010±0.005 | 0.04 | -0.004±0.002 | 0.09 |
| Yellow | 0.015±0.004 | 5.8×10^-5^ | 0.000±0.005 | 0.95 | -0.003±0.002 | 0.23 |
| Pink | 0.010±0.004 | 0.01 | 0.006±0.005 | 0.26 | 0.006±0.003 | 0.02 |
| Red | 0.002±0.004 | 0.62 | -0.009±0.005 | 0.09 | 0.000±0.002 | 0.97 |
| Turquoise | 0.000±0.004 | 0.94 | 0.003±0.006 | 0.53 | 0.002±0.003 | 0.38 |
| Black | 0.011±0.004 | 0.004 | 0.008±0.005 | 0.12 | 0.001±0.003 | 0.67 |
| Brown | 0.003±0.004 | 0.41 | 0.000±0.005 | 0.98 | 0.000±0.003 | 0.96 |
| Green | 0.007±0.004 | 0.08 | -0.003±0.005 | 0.54 | -0.007±0.003 | 0.01 |
| **Non-black** (n= 359 term controls; 18 with mPTD and preeclampsia; 25 with mPTD only; and 71 with sPTD) | | | | | | |
| Blue | -0.006±0.007 | 0.43 | -0.011±0.006 | 0.05 | -0.002±0.004 | 0.66 |
| Yellow | 0.021±0.007 | 0.002 | -0.002±0.006 | 0.76 | -0.004±0.004 | 0.32 |
| Pink | 0.006±0.007 | 0.36 | 0.003±0.006 | 0.65 | 0.000±0.004 | 0.91 |
| Red | -0.002±0.007 | 0.74 | -0.008±0.006 | 0.14 | -0.006±0.004 | 0.10 |
| Turquoise | -0.005±0.007 | 0.47 | -0.001±0.006 | 0.87 | -0.004±0.004 | 0.21 |
| Black | 0.022±0.007 | 7.4×10^-4^ | 0.012±0.006 | 0.03 | 0.004±0.004 | 0.21 |
| Brown | 0.000±0.007 | 0.98 | 0.001±0.006 | 0.88 | 0.001±0.004 | 0.72 |
| Green | 0.023±0.007 | 0.001 | 0.009±0.006 | 0.12 | -0.004±0.004 | 0.24 |

^a^ Compared to women with term delivery, with the adjustment of an array of conventional and clinical factors, including maternal age at delivery, maternal ethnicity/race, maternal birthplace (US-born vs non-US-born), maternal pregestational BMI category, pregestational diabetes, chronic hypertension, marital status, highest education level, smoking during pregnancy, illicit drug use, lifetime stress, and fetal sex.

**Table S5.** Maternal metabolites that were significantly different in women with late sPTD and in women with early sPTD, separately, compared to women with term delivery.

| **Metabolite** | **Classification** | **Any sPTD** | | | **Late sPTD** | | | **Early sPTD** | |
| --- | --- | --- | --- | --- | --- | --- | --- | --- | --- |
|  |  | **Beta±Se^a^** | ***P*** |  | **Beta±Se^a^** | ***P*** |  | **Beta±Se^a^** | **P** |
| Alloisoleucine | Amino Acids | -0.31±0.07 | 2.2×10^-5^ | | -0.12±0.09 | 0.17 | | -0.76±0.12 | 9.0×10^-10^ |
| 3-aminoisobutyrate | Amino Acids | -0.22±0.07 | 0.003 | | -0.04±0.09 | 0.60 | | -0.63±0.12 | 4.3×10^-7^ |
| Hydroxyproline | Amino Acids | -0.15±0.07 | 0.05 | | 0.06±0.09 | 0.46 | | -0.64±0.13 | 4.4×10^-7^ |
| Hippurate | Amino Acids | -0.26±0.08 | 5.7×10^-4^ | | -0.13±0.09 | 0.14 | | -0.60±0.12 | 1.6×10^-6^ |
| N-acetylhistidine | Amino Acids | -0.20±0.07 | 0.008 | | -0.05±0.09 | 0.54 | | -0.51±0.12 | 5.0×10^-5^ |
| Phenylacetylglutamine | Amino Acids | -0.28±0.07 | 1.9×10^-4^ | | -0.21±0.09 | 0.02 | | -0.48±0.12 | 1.2×10^-4^ |
| SDMA | Amino Acids | -0.32±0.08 | 2.7×10^-5^ | | -0.14±0.09 | 0.10 | | -0.73±0.12 | 6.6×10^-9^ |
| C9 carnitine | Acylcarnitine | -0.36±0.07 | 1.4×10^-6^ | | -0.26±0.09 | 0.003 | | -0.57±0.13 | 4.9×10^-6^ |
| Anthranilic acid | Aminobenzoic | -0.33±0.08 | 1.0×10^-5^ | | -0.16±0.09 | 0.06 | | -0.73±0.12 | 5.6×10^-9 b^ |
| Biliverdin | Cofactors and Vitamins | -0.25±0.07 | 8.4×10^-4^ | | -0.14±0.09 | 0.11 | | -0.50±0.12 | 6.1×10^-5^ |
| Bilirubin | Cofactors and Vitamins | -0.44±0.07 | 2.9×10^-9^ | | -0.45±0.08 | 1.5×10^-7^ | | -0.45±0.12 | 2.8×10^-4^ |
| 4-acetamidobutanoate | Fatty Acids | -0.30±0.07 | 4.8×10^-5^ | | -0.18±0.09 | 0.04 | | -0.57±0.12 | 3.8×10^-6^ |
| Acetyl-galactosamine | Monosaccharides | -0.22±0.08 | 0.004 | | -0.09±0.09 | 0.30 | | -0.52±0.13 | 5.1×10^-5^ |
| N-acetylgalactosamine | Monosaccharides | -0.19±0.08 | 0.01 | | -0.05±0.09 | 0.58 | | -0.51±0.13 | 9.2×10^-5^ |
| Adenosine | Nucleotide | -0.27±0.08 | 3.3×10^-4^ | | -0.12±0.09 | 0.17 | | -0.62±0.13 | 8.5×10^-7^ |
| 7-methylguanine | Nucleotide | -0.35±0.07 | 3.6×10^-6^ | | -0.27±0.09 | 0.002 | | -0.54±0.13 | 1.9×10^-5^ |
| Urate | Nucleotide | -0.31±0.07 | 1.7×10^-5^ | | -0.18±0.08 | 0.03 | | -0.61±0.12 | 6.8×10^-7^ |
| Phosphocholine | Quaternary Amines | 0.43±0.08 | 1.2×10^-8^ | | 0.31±0.09 | 4.2×10^-4^ | | 0.72±0.13 | 1.7×10^-8^ |
| Cortisol | Steroid | -0.39±0.07 | 1.6×10^-7^ | | -0.20±0.08 | 0.02 | | -0.83±0.12 | 6.4×10^-12 b^ |
| Cortisone | Steroid | -0.39+0.07 | 1.4×10^-7^ | | -0.12+0.08 | 0.13 | | -1.02+0.11 | 1.3×10^-17 b^ |
| **Lipids** |  |  |  | |  |  | |  |  |
| C18:0 CE | Cholesteryl ester | 0.23±0.07 | 0.002 | | 0.10±0.09 | 0.22 | | 0.51±0.12 | 4.1×10^-5^ |
| C20:3 CE | Cholesteryl ester | 0.17±0.08 | 0.03 | | -0.01±0.09 | 0.95 | | 0.54±0.13 | 2.1×10^-5^ |
| C20:4 CE | Cholesteryl ester | 0.17±0.07 | 0.02 | | 0.01±0.08 | 0.88 | | 0.50±0.12 | 2.7×10^-5^ |
| C22:4 CE | Cholesteryl ester | 0.13±0.07 | 0.07 | | -0.05±0.09 | 0.59 | | 0.52±0.12 | 2.5×10^-5^ |
| C22:5 LPC | Lysophosphatidylcholine | 0.13+0.07 | 0.08 | | -0.03+0.09 | 0.71 | | 0.52+0.12 | 2.7×10^-5 b^ |
| C22:6 LPC | Lysophosphatidylcholine | 0.25±0.07 | 7.6×10^-4^ | | 0.14±0.08 | 0.09 | | 0.53±0.12 | 1.5×10^-5 b^ |
| C34:2 PE | Phosphatidylethanolamine | -0.25±0.07 | 4.8×10^-4^ | | -0.08±0.08 | 0.32 | | -0.62±0.12 | 1.7×10^-7 c^ |
| C36:2 PE | Phosphatidylethanolamine | -0.18±0.07 | 0.01 | | -0.03±0.08 | 0.72 | | -0.50±0.12 | 2.5×10^-5 c^ |
| C36:3 PE | Phosphatidylethanolamine | -0.17±0.07 | 0.02 | | -0.00±0.08 | 0.97 | | -0.54±0.12 | 5.6×10^-6^ |
| C36:2 PI | Phosphatidylinositol | -0.20±0.07 | 0.01 | | -0.03±0.09 | 0.70 | | -0.57±0.12 | 4.9×10^-6^ |
| **Others** |  |  |  | |  |  | |  |  |
| N-ethylglycinexylidide | Other | -0.24±0.07 | 0.001 | | -0.07±0.09 | 0.42 | | -0.63±0.12 | 5.1×10^-7^ |
| Trimethylamine-N-oxide | Other | -0.31±0.07 | 3.5×10^-5^ | | -0.18±0.09 | 0.03 | | -0.60±0.13 | 1.9×10^-6^ |

SDMA: Symmetric dimethylarginine.

^a^ Adjusted for an array of conventional and clinical factors, including maternal age at delivery, maternal ethnicity/race, maternal birthplace (US-born vs non-US-born), maternal pregestational BMI category, pregestational diabetes, chronic hypertension, marital status, highest education level, parity, smoking during pregnancy, illicit drug use, lifetime stress, and fetal sex.

^b^ The metabolite had a similar and significant association with medically-indicated PTD and preeclampsia.

^c^ The metabolite had a similar and significant association with medically-indicated PTD without preeclampsia.

**Table S6.** Associations of metabolite modules with early and late sPTD, separately, compared to women with term delivery.

| Metabolite module | Hub metabolite | Late sPTD (33-36^6/7^ week of gestation)  (n=152) | | | Early sPTD (<33 weeks of gestation)  (n=67) | |
| --- | --- | --- | --- | --- | --- | --- |
|  |  | Beta±Se**^a^** | p |  | Beta±Se**^a^** | p |
| Blue | C44:1 TAG | -0.003±0.002 | 0.26 | | -0.004±0.003 | 0.27 |
| Yellow | C16:0 CE | 0.000±0.002 | 0.88 | | -0.012±0.003 | 0.0003 |
| Pink | C18:1 LPC | 0.003±0.002 | 0.22 | | 0.009±0.003 | 0.0063 |
| Red | C32:2 PC | -0.001±0.002 | 0.72 | | -0.005±0.003 | 0.16 |
| Turquoise | C16:0 SM | 0.000±0.002 | 0.87 | | 0.002±0.004 | 0.55 |
| Black | C14:1Carnitine | 0.002±0.002 | 0.32 | | 0.001±0.003 | 0.82 |
| Brown | Valine | 0.004±0.002 | 0.12 | | -0.005±0.004 | 0.14 |
| Green | Pseudouridine | -0.003±0.002 | 0.16 | | -0.012±0.003 | 0.0003 |

^a^ Adjusted for an array of conventional and clinical factors, including maternal age at delivery, maternal ethnicity/race, maternal birthplace (US-born vs non-US-born), maternal pregestational BMI category, pregestational diabetes, chronic hypertension, marital status, highest education level, parity, smoking during pregnancy, illicit drug use, lifetime stress, and fetal sex
