# Supplementary material for: Postpartum plasma metabolomic profile among women with preeclampsia and preterm delivery: implications for long-term health
Source: BMC Med. 2020 Oct 13;18:277. doi: 10.1186/s12916-020-01741-4 (PMC7552364; doi:10.1186/s12916-020-01741-4)

**Figure S1. Manhattan plot for the metabolomic differences in women with early and late spontaneous PTD, separately, compared to women with term delivery.** The upper panel presents metabolites that were higher, and the lower panel presents metabolites that were lower in women with late sPTD (Figure S1A) and in women with early sPTD (Figure S1B), with adjustment for maternal age at delivery, maternal ethnicity/race, maternal birthplace, maternal pregestational BMI category, pregestational diabetes, chronic hypertension, marital status, highest education level, parity, smoking during pregnancy, illicit drug use, lifetime stress, and fetal sex.


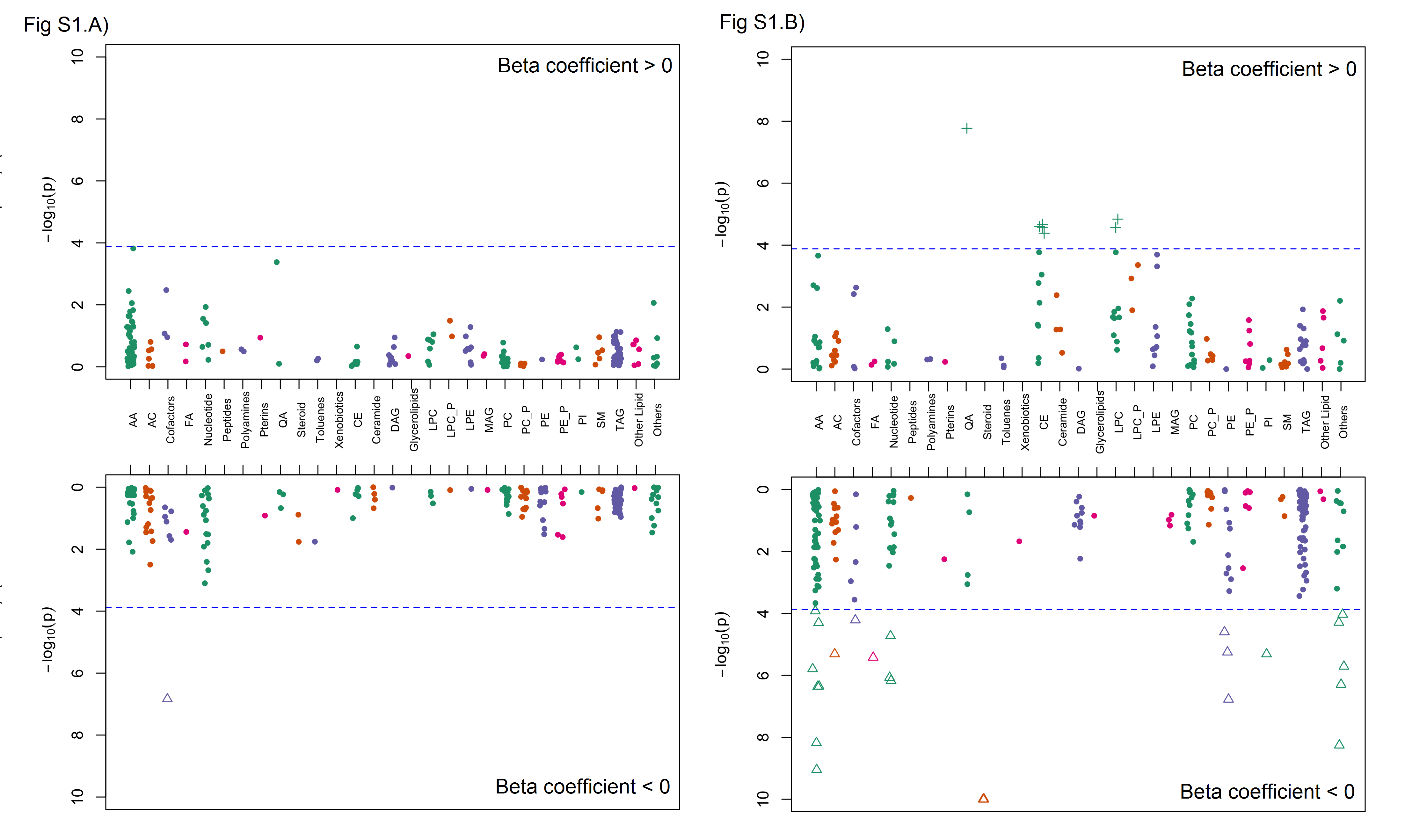

Supplement: Supplementary file 2 — Additional file 2: Figure S1. Manhattan plot for the metabolomic differences in women with early and late spontaneous PTD, separately, compared to women with term delivery. [file 12916_2020_1741_MOESM2_ESM.docx]
